# Use of artificial intelligence within the gambling field: a scoping review protocol

**DOI:** 10.12688/f1000research.167592.2

**Published:** 2026-06-30

**Authors:** Ståle Pallesen, Elise Constance Fodstad, Conchita Sisi Martin, Farangis Sharifibastan, Rune Krumsvik, Hailemariam Weldemariam

**Affiliations:** 1 Department of Psychosocial Science, University of Bergen, Bergen, 5020, Norway; 2 Norwegian Competence Center for Gambling and Gaming Reseach Research, University of Bergen, Bergen, 5020, Norway; 3 Centre for Alcohol and Drug Research (KORFOR), Stavanger University Hospital, Stavanger, Rogaland, Norway; 4 Department of Pscyhology, Camilo Jose Cela University, Villafranca del Castillo, Community of Madrid, Spain; 5 Department of Education, University of Bergen, Bergen, 5020, Norway; 6 Human Enhancement and Body Image Lab (HEBI Lab), University of Bergen, Bergen, 5020, Norway

**Keywords:** artificial intelligence, machine learning, gambling, scoping review, protocol.

## Abstract

**Introduction:**

This scoping review aims to map existing studies that have employed artificial intelligence (AI) tools within the gambling field, examining their areas of use, current trends, and key findings.

**Methods and analysis:**

This review will adhere to the Joanna Briggs Institute Reviewers’ Manual. The review will be organized along the Population, Concept and Context approach. It will include quantitative peer-reviewed studies that examine the use of AI tools within gambling contexts. Searches for relevant articles will be conducted in Web of Science, APA PsycINFO, Medline (Ovid), ProQuest, CINAHL, and Wiley Online Library. A search for grey literature will be conducted in GreyLit. Org, ProQuest Dissertations and Theses, Google Scholar, and Google search engine, reviewing the first 50 results in Incognito mode. Two independent reviewers will perform screening, selection, and data extraction, with disagreements resolved through discussion or consultation with a third reviewer. The results will be presented in graphical and tabular format, accompanied by a narrative summary following the PRISMA-ScR guidelines. The protocol has been pre-registered in Open Science Framework: https://doi.org/10.17605/OSF.IO/FMBE6

**Ethics and dissemination:**

This study protocol is exempted from ethical approval. The planned review aims to describe how AI has been used within the gambling field and has as such as a goal to inform various stakeholders such as clinicians, gambling operators as well as regulatory authorities. The scoping review will be published in an open access journal.

## Introduction

Gambling involves staking money or other valuables on the outcome of a game or event determined, partially or entirely, by chance. ^1^ Today, gambling activities occur in the vast majority of cultures and can be traced back 4,000 years. ^2^ Prevalence studies indicate that almost half of adults worldwide have gambled in the past 12 months. ^3^ Globally, it is estimated that 8.7% of adults engage in *risky gambling*, while 1.4% struggle *with problematic gambling.* ^3^ The diagnosis of *gambling disorder* is reserved for the most severe cases and is characterized by impaired control over gambling, an increasing priority given to gambling, and the continuation or escalation of gambling despite negative consequences. ^4^

The global gambling market was valued at approximately $540 billion in 2023, and is expected to grow 6.4% annually, driven by factors such as legalization, urbanization, increased social media use, and a rising population. ^5^ Various technological advancements are anticipated to spur growth in online gambling channels, including gambling apps and online casinos. ^5, 6^ *Responsible gambling* initiatives aim to promote awareness and prevent harms associated with gambling, but many of these have faced criticism for placing one onus of responsibility on the individuals. ^7^ Wardle et al. ^8^ argue that the growth of the gambling industry is facilitated by strong ties to governments, and emphasize that health related harms of problematic gambling should take precedence over economic interests, commercial profitability, and government revenue generation. Online gambling is expanding rapidly, and in parallel so does the amount of player account data, ^9^ which facilitates the use of various *artificial intelligence* (AI) technologies. ^10^

AI refers to computer systems that automatically perform complex tasks such as perceiving, reasoning, decision making, problem-solving, and learning, which require intelligence when conducted by humans. ^11– 13^ Some AI technologies mine data, learn, recognize speech and images, and analyze cognitions and emotions. ^11^ *Machine learning* involves computer algorithms that identify patterns in data and enhance their performance automatically over time. ^14, 15^ Among machine learning subsets, *deep learning* employes a group of machine learning algorithms to execute high-level abstractions in data, resembling human brain functions, using deep architectures or interconnected nodes that constitute multiple non-linear transformations. Deep learning systems automatically learn features at multiple levels of abstraction, enabling them to learn complex functions and map raw sensory input data to the output without human intervention. ^16^ Deep learning techniques can be applied in areas such as speech recognition, ^17^ object recognition, ^18^ and natural language processing. ^19^ On the other hand, *artificial neural networks*, which resemble biological neural networks, perform specific tasks similarly to that of the human brain, using hundreds or thousands of interconnected artificial neurons or nodes that process and transmit information. ^20, 21^ AI has revolutionized automated online service interactions through advancement in algorithms, massive data and affordable computational power and storage. ^22^ By transforming online consumer behavior into actionable strategies, AI-based digital market businesses can access their customers just-in-time. ^23– 25^ Features like *speech recognition* and machine learning, enhance mixed reality (MR), immersion, enjoyment and consumers’ perception, positively impacting engagement, purchase intentions and social sharing. ^26^ Studies also highlight AI’s ability to strengthen relationships between digital markets and online users. ^27, 28^

In the realm of gambling, AI systems collect and process various forms of consumer data in real time to enhance the understanding of customers (Dos Santos, 2015). For example, models based on neural networks have been developed to predict bet amounts and cumulative winnings/losses. ^29^ Several studies have used AI to identify problem gamblers. ^30– 33^ In one study, Auer and Griffiths ^34^ used objective account based data from online casino players and matched it with self-reported gambling problems. Based on data such as wagering, depositing, and gambling frequency, random forest and gradient boost machine algorithms were trained to predict self-reported problem gambling. The two AI algorithms achieved an area under the curve (AUC) value of.73 and.67, respectively, which is considerably better than chance. ^34^ Other studies using machine learning models have exclusively used account based data, showing that early behaviors (within the first 7 days) can predict later high risk gambling. ^35^ Machine learning models, exclusively based on survey data have shown that reports of gambling problems can be predicted by various demographic and self-reported gambling-related behaviors. ^36^ Suicidal ideation and suicide attempts among gamblers have also been modelled with machine learning using similar data. ^37^ In addition, AI has also been used to identify personality traits that distinguish between gambling disorder patients and healthy controls. ^38^ One study used mixed logistic regression machine learning based on data from four bet types and eight seasons of English Premier League soccer, finding that losses were positively correlated with observable betting odds. The authors suggest incorporating differences in product risk into consumer education and responsible gambling approaches. ^39^ Furthermore, Large Language Models (LLM) have, for example, been utilized to respond to prompts regarding problem gambling. In this capacity, they can assist individuals in overcoming barriers (e.g., stigma) to seeking help, by offering an anonymous and judgment-free platform. ^40^ LLM also hold promise in detecting gambling issues within various online texts. ^41^

Another application of AI within the gambling sphere concerns predicting outcomes in skill-based games (e.g., poker). Studies have shown that AI can outperform top poker players in some cases. ^42, 43^ Research has also been conducted to assess and validate the skills of poker players against AI-simulated games. ^44^ A qualitative interview study with gambling industry stakeholders suggests that while AI holds promise for consumer protection, there is also an inherent risk of increased player exploitation (e.g., creating more addictive games and more persuasive marketing tactics). ^45^

Based on this backdrop, the aim of this scoping review is to map studies where AI-based tools have been used within the gambling field, areas of use as well as current trends and findings.

### Protocol

We decided to conduct a scoping review on the topic of AI use within the gambling field as both fields recently have undergone rapid changes. The use of AI seems to explode within many sectors. ^46^ Parallelly, gambling is moving towards digital and online games. ^47^ This enables AI to have a large impact on the emerging gambling field. Cardoso and colleagues published a systematic review focusing on how AI can be used to promote safer gambling experiences, ^48^ whereas Binesh and Ghaharian recently reviewed risks and ethical considerations of AI in gambling. ^49^ Although reviews such as those referred to above have been published, a broader review on AI within the gambling field is missing. We therefore found it timely to broadly and systematically map the use of AI within this domain by employing a scoping review approach. This review will be conducted following JBI’s scoping review methodology ^50^ and reported using the Preferred Reporting Items for Systematic Reviews and Meta-Analyses extension for Scoping Reviews; PRISMA-ScR) guidelines. ^51^ It was not deemed appropriate or possible to involve patients or the public in the design, or conduct, or reporting, or dissemination plans of our research as the research concerns a literature review.

### Review questions

The aim of the review is to provide an overview of how and for which purposes AI is used within the gambling field and describe various AI-models used, trends thereof and the main empirical findings from the identified studies. This lead to the development of the following research questions: 1) How are AI-technology and AI-tools used (purposes) within the gambling field?, and 2) What characterizes the AI-models used, what are the trends in AI technology use, and what are the main findings?

### Inclusion and exclusion criteria

The inclusion and exclusion criteria will follow the participants-concept-context model outlined by Peters et al. ^50^

**Participants.** No specific criteria regarding participants will be applied in the current study.

**Concept.** The central concept concerns the use of AI-tools within the gambling field. These tools refer to computer systems that automatically accomplish complex tasks, such as reasoning and decision making, which require intelligence when conducted by humans. Specifically, these tools encompass machine learning, deep learning, reinforcement learning, neural networks, natural language processing, supervised and unsupervised learning, decision trees, probabilistic models, Bayesian network, extreme gradient boosting, long short-term memory, generative adversarial networks, CatBoost, and random forests.

**Context.** Gambling implies staking money on a future outcome that is at least partly determined by chance. Eligible studies will broadly cover the use of AI-tools broadly within the field of gambling, and address issues such as identifying specific groups of gamblers (e.g., problem gamblers), and modelling human behavior and decision making in gambling, often where skills are involved. Studies on gamblers’ attitudes towards AI-incorporated features in gambling will not be eligible as these do not relate to the actual use of AI.

### Type of sources

This review will mostly include quantitative peer-reviewed studies where AI-tools have been used in the context of gambling, alongside selected grey literature. Reviews and qualitative studies will not be included. Studies will be sourced from scientific databases (see later) and journals (backwards tracking). Abstracts, and theses will not be included. Only articles written in English will be considered.

### Search strategy

The search strategy will follow a three-step approach as outlined in the JBI Manual for Evidence Synthesis for Scoping Reviews. ^50^

1. **Initial search:** A targeted search will be conducted in APA PsycINFO, Web of Science, and Google Scholar to identify relevant studies and refine the search terms. The search string will include the concept term “artificial intelligence” and relevant subcategories (“machine learning”, “deep learning”, “reinforcement learning”, “neural networks”, “natural language processing”, “NLP”, “supervised learning”, “unsupervised learning”, “decision trees”, “probabilistic models”, “Bayesian network”, “Extreme Gradient Boosting”, “XGBoost”, “Generative Adversarial Network”, “GANs”, “CatBoost”, and “Random Forest”) combined with the term gambl*. Boolean operators will be used, with “OR” connecting related terms within each group/category and “AND” linking the two main categories (AI and gambling). A preliminary search covering 2025 and 2026, adapted to the specific characteristics of APA PsycINFO, Web of Science, and Google Scholar, was conducted in order to identify additional keywords and index terms (see Table 1).
2. **Refined search strategy:** The refined search strategy will be applied to additional databases to ensure comprehensive coverage. These databases will include Medline (Ovid), ProQuest, CINAHL, and Wiley Online Library. Given the relatively recent emergence of this topic, we have decided not to impose any time restrictions on articles for inclusion. The search is restricted to English-language publications, as most relevant studies and AI methodologies are reported in English. Including additional keywords and indexing terms from the initial search will enhance the breadth and accuracy of the refined strategy. Boolean operators will be used again to maximize the retrieval of relevant studies across databases. For exclusion criteria, articles referring to gaming (e.g., video gaming or e-sports) will not be considered, as the focus of this review is specifically on gambling-related contexts.
3. **Identifying additional studies:** Manual screening of reference lists from included articles and searches of grey literature will be conducted. Grey literature searches will cover sources such as GreyLit. Org, ProQuest Dissertations and Theses, Google, and Google Scholar, with the first 50 results reviewed in Incognito mode to reduce bias from browsing history. The search results, including records retrieved, screened, and included, will be documented and visualized using the PRISMA flow diagram. ^52^ In terms of grey literature, only conference proceedings and dissertations will be considered due to the emerging nature of the topic. Other types of publications will be excluded as they do not meet the necessary quality standards. Included grey literature will be assessed using the ACCOODS Checklist covering the following dimensions: Authority, accuracy, coverage, objectivity, date, and significance. ^53^

**Table 1.** Draft search strategy.

Database	Query	Records retrieved
Web of Science	gambl* AND ((artificial intelligence) OR (machine learning) OR (deep learning) OR (reinforcement learning) OR (neural networks) OR (natural language processing) OR (NLP) OR (supervised learning) OR (unsupervised learning) OR (decision trees) OR (probabilistic models) OR (Bayesian network) OR (Extreme Gradient Boosting) OR (XGBOOST) OR (Large Language Model) OR (LLM) OR (Generative Adversarial Network) OR (GANS) OR (CatBoost) OR (Random Forest) OR (Tree-based algorithms) OR (ChatGPT) OR (Long Short-Term Memory) OR (Gated Recurrent Unit)	388

Web of Science.

Search conducted on January 17, 2025.

Language filters (English) were applied.

### Selection of papers

The Covidence Systematic Review software ^54^ will be used to manage references throughout the review process. All citations identified through the search process will be uploaded to Covidence for duplicate removal and initial screening by title and abstract. A pilot screening will be performed on 10 of the identified citations by two independent reviewers (CSM and FS) to ensure consistency in applying the eligibility criteria. Following this, a consensus meeting will be held to discuss the pre-defined inclusion and exclusion criteria, with adjustments made if necessary. After the pilot screening, the remaining citations will be screened by title and abstract in line with the eligibility criteria. Screening will be conducted by two independent reviewers (CSM and FS), and studies deemed eligible will be advanced for full-text review. In the full-text review stage, two independent reviewers (CSM and FS), will evaluate all selected studies. Reasons for excluding studies during this phase will be systematically documented and included in the final report. Any disagreements between reviewers during the selection process will be resolved through discussion and consensus with a third reviewer (SP). The search results and study selection process will be detailed and presented in a PRISMA flow diagram, ^52^ ensuring transparency and adherence to scoping review reporting standards.

### Data extraction

Data extraction will follow the strategy of “data charting” described in the JBI template. ^50^ We developed a draft charting form that will be adjusted after the pilot extraction (See Table 2). We will extract data on authorship, year and type of publication, country of origin, study objective, methodology, population, AI characteristics, type of gambling, and key findings. The studies will be categorized according to our review question.

**Table 2.** Draft data charting form.

**Publication details**
Author(s)
Year of publication
Type of publication
Country of origin
Language
Aims/purpose/objective
Population and sample size
Methodology/design
**AI and gambling details**
AI characteristics
-AI model
-Model complexity (number of parameters, features and interactions in algorithms)
-Algorithms
-Type of supervision (technical, e.g., based on historical data vs. human involved)
-Task performed
-Availability of source code
-Ethics (e.g., privacy, algorithmic bias, or player exploitation)
Gambling characteristics (e.g., online/offline, lottery)
Categorizations regarding our review questions ^1^
Key findings (e.g., AUC, accuracy, precision)

Note: Categories may be adjusted following pilot extraction.

During the pilot extraction, all reviewers will independently extract data from the same 10 papers. A meeting with all reviewers will then serve to calibrate the extraction process and adjust the charting form. Further extraction will be done by two independent reviewers (CSM and FS). Disagreements will be resolved in a discussion meeting with the two reviewers, and a third reviewer (SP) will be involved if agreement is not achieved.

### Data analysis, presentation and dissemination

Search strategy and study selection will be reported following the PRISMA flowchart. ^52^ Results will be presented in tabular (and graphical) format, along with a narrative summary. Descriptive statistical reporting frequencies will be presented. The plan includes utilizing frequency diagrams to illustrate the types of AI models and gambling-related themes. Furthermore, the frequency of technical versus human-involved supervision, alongside specific types of gambling, will be quantified and presented. The presentation will follow the recommendations by the Preferred Reporting Items for Systematic Reviews and Meta-Analyses extension for Scoping Reviews (PRISMA-ScR). ^51^ We will prepare a paper for submission to an open-access journal. The paper will present both risk-detection models, highly relevant for clinicians, and predictive behavioral modeling, which is of particular interest to regulators. The final data will be shared on the OSF.

## Discussion

AI is used increasingly within the gambling industry. Based on both subjective data as well as player account data AI holds promise as a tool for predicting future gambling behavior, identifying those with problems as well as validating specific skills involved in gambling. Within the gambling context AI may thus be beneficiary for customers, however the potential for misuse in terms of customer exploitation cannot be dismissed. We regard the scoping review approach as the best suited method to gain an overview and to make a synthesis regarding how AI currently is being used within the gambling industry as well as pointing out future and coming trends. The review will inform various stakeholders (e.g., regulatory agencies, the industry, clinicians as well as various user organization) about the use of AI within the gambling field. A limitation of the planned scoping review is that it will be restricted to publications in English. In addition, unpublished paper/report made by the industry will not be included. To ensure the scientific rigor of the studies included in this scoping review, grey literature will be restricted to conference proceedings and dissertations. Still, this may leave out some relevant publications. Qualitative studies will be excluded as the review aims to map quantifiable outcomes, statistical correlations, and numerical trends that fall outside the scope of qualitative methodologies. Unlike previous reviews on the topic, ^48, 49^ this review will provide a broad and comprehensive overview of AI applications within the gambling field. Additionally, it aims to highlight current methodological shortcomings and will suggest directions for future research, while discussing the implications for operators, clinicians, and regulators, respectively.

## Data Availability

No data is associated with this article.
