# Supplementary material for: Use of artificial intelligence within the gambling field: a scoping review protocol
Source: F1000Res. 2026 Jun 30;14:807. Originally published 2025 Aug 20. [Version 2] doi: 10.12688/f1000research.167592.2 (PMC13570114; doi:10.12688/f1000research.167592.2)
Supplement: Peer review history

# Reviewer report: Reviewer response for version 2

**DOI:** 10.5256/f1000research.203773.r497860

**Published:** 2026-09-11

**Authors:** Robert Heirene

**Affiliations:** 1 Brain & Mind Centre, School of Psychology, Science Faculty, The University of Sydney, Sydney, New South Wales, Australia

The authors have addressed my initial concerns and I have no further comments. I look forward to seeing the final, indexed review paper.

Is the study design appropriate for the research question?

Yes

Is the rationale for, and objectives of, the study clearly described?

Partly

Are sufficient details of the methods provided to allow replication by others?

Partly

Are the datasets clearly presented in a useable and accessible format?

Not applicable

Reviewer Expertise:

I have a background in psychology and the study of addictive disorders. My research primarily focuses on using the behavioural data collected by gambling operators to understand markers of harmful gambling and to evaluate the impact of harm-prevention interventions and policies.

I confirm that I have read this submission and believe that I have an appropriate level of expertise to confirm that it is of an acceptable scientific standard.

# Reviewer report: Reviewer response for version 2

**DOI:** 10.5256/f1000research.203773.r497861

**Published:** 2026-07-22

**Authors:** René Manassé Galekwa

**Affiliations:** 1 University of Klagenfurt, Klagenfurt, Austria

I thank the authors for their careful and constructive responses to my comments. The authors have clearly strengthened the revised protocol, and they have satisfactorily addressed most of my recommendations. I confirm the following points as resolved:

1. **Data extraction - ethical dimensions.** The dedicated field capturing whether studies discuss ethics (privacy, algorithmic bias, and player exploitation) has been added to Table 2. This information is a valuable addition given the ethical paradox the introduction rightly foregrounds.
2. **Data extraction - performance metrics.** The specification of performance data (AUC, accuracy, precision) under key findings is now explicit and will support a more robust final synthesis.
3. **Dissemination and impact.** The tailoring of findings to different stakeholder groups—risk-detection models for clinicians and predictive behavioral modeling for regulators—is now clearly articulated.
4. **Exclusion of qualitative research.** The rationale added to the discussion is clear and appropriate.
5. **Limitations of the grey literature search.** The acknowledgment that restricting grey literature to conference proceedings and dissertations may omit relevant publications is a welcome addition.

One point from my original review remains only partially addressed, and I would ask the authors to revisit it:

**Categorization of LLMs is presented in the data charting form.** My original recommendation had two parts: (a) a brief discussion of how LLMs are expected to appear in the gambling sector, and (b) how these tools will be *categorized within the data charting form (Table 2)*. The authors have addressed (a) well by expanding the introduction, but they have not yet answered the operational question (b). It remains unclear how an LLM-based study would be recorded under the current "AI model" and "algorithms" fields—for example, whether LLMs will be treated as a distinct model category, nested under natural language processing, or handled some other way. Because LLM-based applications differ substantially from the tabular/behavioral models that dominate the existing literature (in architecture, parameter scale, and evaluation metrics), a brief note in the methods or a coding convention for Table 2 would improve extraction consistency between reviewers and make the eventual synthesis clearer. I would encourage the authors to add a sentence or two specifying the details.

 With this final clarification, I would be pleased to see the protocol approved.

Is the study design appropriate for the research question?

Yes

Is the rationale for, and objectives of, the study clearly described?

Yes

Are sufficient details of the methods provided to allow replication by others?

Yes

Are the datasets clearly presented in a useable and accessible format?

Not applicable

Reviewer Expertise:

I am a PhD candidate and research student specializing in Artificial Intelligence. My research focuses on using machine learning techniques to predict sporting events, manage betting risks, and optimize bettor portfolios.

I confirm that I have read this submission and believe that I have an appropriate level of expertise to confirm that it is of an acceptable scientific standard.

# Reviewer report: Reviewer response for version 1

**DOI:** 10.5256/f1000research.184717.r450590

**Published:** 2026-02-27

**Authors:** Robert Heirene

**Affiliations:** 1 Brain & Mind Centre, School of Psychology, Science Faculty, The University of Sydney, Sydney, New South Wales, Australia

Dear Authors & Editorial Team,

 Many thanks for the invitation to review this interesting article. It outlines a protocol for a scoping review of artificial intelligence use cases within the gambling field.

 Please see my comments on each of the sections below.

**Introduction**

 Third paragraph: “Some AI’s technologies mine data, …” – this seems like a typo to me. Perhaps it should read “Some AI technologies mine data, …”

 Overall, the introduction sets the scene for this review reasonably well, but the focus seems to be almost exclusively on AI for risk detection, with other use cases only briefly mentioned at the end. There is no mention of large language models, despite the rapid growth in their use in several domains, including gambling (see: Ref 1); although I note that this term is included in the search strategy, which is positive. Second, at least part of the rationale for the review is that there is a “lack of current views on the topic”. A very similar review was published recently on this topic, and so the author should be careful to state how their review will differ from this previous article and why their review is necessary:

- Cardoso, L. G., Barroso, B. C. R., Piccoli, G., Peixoto, M., Morgado, P., Marques, A., Rocha, C., Griffiths, M. D., Queirós, R., & Dores, A. R. (2026). Deep technologies and safer gambling: A systematic review. *Acta psychologica*, *262*, 106140. https://doi.org/10.1016/j.actpsy.2025.106140

Another similar review the authors should beware of is:

- Binesh, F., & Ghaharian, K. (2025). Identifying Risks and Ethical Considerations of AI in Gambling: A Scoping Review. *International Journal of Hospitality & Tourism Administration*, 1–31. https://doi.org/10.1080/15256480.2025.2494575

**Methods**

 Eligibility criteria: Is there a reason why only quantitative studies will be included? A qualitative study is referenced at the end of the introduction, which could be highly informative for this review.

 Eligibility criteria & search strategy: The authors state that only peer-reviewed studies will be included, but then state that they will search grey literature sources like theses databases and conference proceedings (which may or may not have been peer-reviewed). Please clarify this inconsistency.

 Search strategy: Is there any reason why the initial/pilot search has not already been conducted and the results integrated into the methods proposed here?

 Search strategy: In section 3, the authors state that the “adequacy of the selected publications will be assessed using the ACCOODS Checklist for grey literature” – what does “adequacy” mean here? Also, the cited reference links to a Flinders University page that does not contain any mention of a ACCOODS Checklist. Searching for these terms together on the Flinders site or just “ACCOODS” does not return any results. Please provide a direct link to the source.

 Data extraction: The data charting form provided seems acceptable but basic. I would strongly suggest that the authors provide guidance and/or examples for the more complicated items (e.g., model complexity, type of supervision) to ensure consistency between extractors and across studies.

 Data analysis, presentation and dissemination: “A paper will be produced and sought published in an open access journal.” – removing the word “sought” from this sentence would make it clearer.

 Data analysis, presentation and dissemination: Will there be any quantitative synthesis of outcomes? Even summary figures like the number of papers on each topic? I’d like to see the authors give more thought to how they will synthesise the results and outline their strategy here.

**Discussion/Conclusions**

 There is no discussion or conclusion section. It will be helpful for the authors to include some final comments on the value of this proposed review and what it will contribute to understanding/practice/policy.

**Transparency**

 Can the authors please confirm that they will share the data extracted from the reviewed studies on an openly available repository? Apologies if this is included and I missed it.

**Overall comments**

 The proposed review sounds interesting, but the authors have not fully explained how it will benefit the field above and beyond what has already been published recently in the literature. Further, many of the methodological details are not clearly outlined. I understand that they plan to pilot test the search strategy and extraction form and then revise these elements of the methodology, but I do not see any reason why this can be done at this stage and included in this protocol. I would expect to see extensive detail of the proposed methodology here to justify a peer-reviewed publication.

 I hope the authors find my comments useful in revising their manuscript.

Is the study design appropriate for the research question?

Yes

Is the rationale for, and objectives of, the study clearly described?

Partly

Are sufficient details of the methods provided to allow replication by others?

Partly

Are the datasets clearly presented in a useable and accessible format?

Not applicable

Reviewer Expertise:

I have a background in psychology and the study of addictive disorders. My research primarily focuses on using the behavioural data collected by gambling operators to understand markers of harmful gambling and to evaluate the impact of harm-prevention interventions and policies.

I confirm that I have read this submission and believe that I have an appropriate level of expertise to confirm that it is of an acceptable scientific standard, however I have significant reservations, as outlined above.

## References

[1] : Can Large Language Models Address Problem Gambling? Expert Insights from Gambling Treatment Professionals. *Journal of Gambling Studies* .2025; 10.1007/s10899-025-10430-x 10.1007/s10899-025-10430-x 41071452

[2] : Deep technologies and safer gambling: A systematic review. *Acta Psychologica* .2026;262: 10.1016/j.actpsy.2025.106140 10.1016/j.actpsy.2025.106140 41601102

[3] : Identifying Risks and Ethical Considerations of AI in Gambling: A Scoping Review. *International Journal of Hospitality & Tourism Administration* .2025; 10.1080/15256480.2025.24945751-31 10.1080/15256480.2025.2494575

# Response

**Published:** 2026-06-17

**Authors:** Ståle Pallesen

**Affiliations:** Dept of Psychosocial Science, University of Bergen, Bergen, Norway

**Dear Authors & Editorial Team,**

 Many thanks for the invitation to review this interesting article. It outlines a protocol for a scoping review of artificial intelligence use cases within the gambling field.

 Please see my comments on each of the sections below.

 Introduction

 Third paragraph: “Some AI’s technologies mine data, …” – this seems like a typo to me. Perhaps it should read “Some AI technologies mine data, …”

**RESPONSE:** Thank you so much for detecting this. The typo has now been corrected.

 Overall, the introduction sets the scene for this review reasonably well, but the focus seems to be almost exclusively on AI for risk detection, with other use cases only briefly mentioned at the end. There is no mention of large language models, despite the rapid growth in their use in several domains, including gambling (see: Ref 1); although I note that this term is included in the search strategy, which is positive.

**RESPONSE:**

 Thanks for this comment, which parallels a comment from reviewer 1. The planned review will not only focus on AI for risk detection, which we hope our introduction and research questions do indicate. The introduction has now been updated regarding LLM and the following text has been added: «Furthermore, Large Language Models (LLM) have, for example, been utilized to respond to prompts regarding problem gambling. In this capacity, they can assist individuals in overcoming barriers (e.g., stigma) to seeking help, by offering an anonymous and judgment-free platform. ^40^ LLM also hold promise in detecting gambling issues within various online texts. ^41^»

**Second,** at least part of the rationale for the review is that there is a “lack of current views on the topic”. A very similar review was published recently on this topic, and so the author should be careful to state how their review will differ from this previous article and why their review is necessary:

- Cardoso, L. G., Barroso, B. C. R., Piccoli, G., Peixoto, M., Morgado, P., Marques, A., Rocha, C., Griffiths, M. D., Queirós, R., & Dores, A. R. (2026). Deep technologies and safer gambling: A systematic review. *Acta psychologica*, *262*, 106140. https://doi.org/10.1016/j.actpsy.2025.106140

Another similar review the authors should beware of is:

- Binesh, F., & Ghaharian, K. (2025). Identifying Risks and Ethical Considerations of AI in Gambling: A Scoping Review. *International Journal of Hospitality & Tourism Administration*, 1–31. https://doi.org/10.1080/15256480.2025.2494575

**RESPONSE:** Thanks for bringing these reviews to our attention. At the time of submission these reviews had not been published. Still, we agree that they definitely should be referred to. We have now, in this regard, added the following text: «Cardoso and colleagues published a systematic review focusing on how AI can be used to promote safer gambling experiences, ^48 ^ whereas Binesh and Ghaharian recently reviewed risks and ethical considerations of AI in gambling. ^4^ ^9^ Further, the text indicating that the present protocol paper refers to the first review on this topic is therefore amended and now reads as follows: “Although reviews such as those referred to above have been published, a broader review on AI within the gambling field is missing. We therefore found it timely to broadly and systematically map the use of AI within this domain by employing a scoping review approach.”

 Methods

 Eligibility criteria: Is there a reason why only quantitative studies will be included? A qualitative study is referenced at the end of the introduction, which could be highly informative for this review.

**RESPONSE:** The following has been added to the discussion in order to explain why qualitative studies will be excluded: «Qualitative studies will be excluded as the review aims to map quantifiable outcomes, statistical correlations, and numerical trends that fall outside the scope of qualitative methodologies.”

 Eligibility criteria & search strategy: The authors state that only peer-reviewed studies will be included, but then state that they will search grey literature sources like theses databases and conference proceedings (which may or may not have been peer-reviewed). Please clarify this inconsistency.

**RESPONSE:** We apologize for this inconsistency. We have now updated the text regarding source types to read: «This review will mostly include quantitative peer-reviewed studies where AI tools have been used in the context of gambling, alongside selected grey literature.»

 Search strategy: Is there any reason why the initial/pilot search has not already been conducted and the results integrated into the methods proposed here?

**RESPONSE:** No, and the search has now been conducted. The text in question has consequently been updated and reads as follows: «A preliminary search covering 2025 and 2026, adapted to the specific characteristics of APA PsycINFO, Web of Science, and Google Scholar, was conducted in order to identify additional keywords and index terms (see Table 1).” A total of four search expressions were added (Tree-based algorithms, ChatGPT, Long Short-Term Memory and Gated Recurrent Unit). Additionally, the topic concerning ethics popped up in the initial search – although this was already mentioned by the two reviewers and consequently added as an AI related topic in the data charting form (table 2).

 Search strategy: In section 3, the authors state that the “adequacy of the selected publications will be assessed using the ACCOODS Checklist for grey literature” – what does “adequacy” mean here? Also, the cited reference links to a Flinders University page that does not contain any mention of a ACCOODS Checklist. Searching for these terms together on the Flinders site or just “ACCOODS” does not return any results. Please provide a direct link to the source.

**RESPONSE:** In terms of the ACCOODS, the text has now been updated so that it now reads: «Included grey literature will be assessed using the ACCOODS Checklist, covering the following dimensions: Authority, accuracy, coverage, objectivity, date, and significance.” We apologize for the erroneous link. The correct link to the ACCOODS Checklist has now been provided (reference updated).

 Data extraction: The data charting form provided seems acceptable but basic. I would strongly suggest that the authors provide guidance and/or examples for the more complicated items (e.g., model complexity, type of supervision) to ensure consistency between extractors and across studies.

**RESPONSE:** We have now added the following to table 2 to provide more details about model complexity «(number of parameters, features and interactions in algorithms)», and the following in terms of Type of supervision: «(technical, e.g., based on historical data vs. human involved).»

 Data analysis, presentation and dissemination: “A paper will be produced and sought published in an open access journal.” – removing the word “sought” from this sentence would make it clearer.

**RESPONSE:** The sentence has now been somewhat rewritten and the word «sought» has been deleted.

 Data analysis, presentation and dissemination: Will there be any quantitative synthesis of outcomes? Even summary figures like the number of papers on each topic? I’d like to see the authors give more thought to how they will synthesise the results and outline their strategy here.

**RESPONSE:** We agree and have added the following to the text: «The plan includes utilizing frequency diagrams to illustrate the types of AI models and gambling-related themes. Furthermore, the frequency of technical versus human-involved supervision, alongside specific types of gambling, will be quantified and presented.”

 Discussion/Conclusions

 There is no discussion or conclusion section. It will be helpful for the authors to include some final comments on the value of this proposed review and what it will contribute to understanding/practice/policy.

**RESPONSE:** The following have now been added to the discussion: «Unlike previous reviews on the topic, ^48, 49^ this review will provide a broad and comprehensive overview of AI applications within the gambling field. Additionally, it aims to highlight current methodological shortcomings and will suggest directions for future research, while discussing the implications for operators, clinicians, and regulators, respectively.”

 Transparency

 Can the authors please confirm that they will share the data extracted from the reviewed studies on an openly available repository? Apologies if this is included and I missed it.

**RESPONSE:** Yes, and the following has now been added to the Data analysis, presentation and dissemination section: «The final data will be shared on the OSF.”

 Overall comments

 The proposed review sounds interesting, but the authors have not fully explained how it will benefit the field above and beyond what has already been published recently in the literature. Further, many of the methodological details are not clearly outlined. I understand that they plan to pilot test the search strategy and extraction form and then revise these elements of the methodology, but I do not see any reason why this can be done at this stage and included in this protocol. I would expect to see extensive detail of the proposed methodology here to justify a peer-reviewed publication.

**RESPONSE:** Pilot testing has now been conducted. The text in question has consequently been updated and reads as follows: «A preliminary search covering 2025 and 2026, adapted to the specific characteristics of APA PsycINFO, Web of Science, and Google Scholar, was conducted in order to identify additional keywords and index terms (see Table 1).” A total of four search expressions were added (Tree-based algorithms, ChatGPT, Long Short-Term Memory, and Gated Recurrent Unit).  Additionally, the topic concerning ethics popped up – although this was already mentioned by the two reviewers and consequently added as an AI related topic in the data charting form (table 2).

 I hope the authors find my comments useful in revising their manuscript.

**REPONSE:** Absolutely. Thank you so much for reviewing the paper and for your constructive and helpful feedback��

# Reviewer report: Reviewer response for version 1

**DOI:** 10.5256/f1000research.184717.r438633

**Published:** 2026-01-07

**Authors:** René Manassé Galekwa

**Affiliations:** 1 University of Klagenfurt, Klagenfurt, Austria

This protocol sets the stage for a scoping review of AI applications within the gambling sector. As the authors point out, AI is already a staple of the digital gambling world—used for everything from harm detection to game design—yet we still lack a clear overview of its methodologies and ethical implications.

 The study is well-structured and follows established PRISMA-ScR and JBI frameworks. With a transparent search strategy and a pre-registered design, the methodology is both rigorous and reproducible. This work provides a much-needed foundation for anyone looking to understand the intersection of AI and gambling, from operators to policy makers.

**Critical Recommendations for Scientific Soundness**

 To further strengthen the protocol and the subsequent review, I suggest addressing the following points:

**1. Defining the scope of "AI Tools"** The inclusion criteria are quite thorough, but the mention of Large Language Models (LLMs) warrants more specific attention. Given the rapid pace of development in 2025, it would be helpful if the authors briefly discussed how they expect LLMs to appear in the gambling sector, perhaps in customer service bots or the analysis of player interactions. Specifically, how will these be categorized in the data charting form?

**2. Enhancing the Data Extraction Plan (Table 2)**

**-Ethical Dimensions:** I recommend adding a dedicated field to Table 2 to capture whether the studies actually discuss ethics (e.g., privacy, algorithmic bias, or player exploitation). This is vital, especially since the introduction already highlights the ethical paradox of AI in this field.

**-Performance Metrics:** To make the "key findings" more useful, the authors should ensure they consistently extract performance data like AUC, accuracy, or precision. This will make the final synthesis much more robust.

**-Refining Dissemination and Impact** While the dissemination plan is good, its impact could be improved by specifying how the findings will be tailored to different groups. For instance, clinicians might need a focus on risk-detection models, while regulators will be more interested in predictive behavioral modeling.

**3. Recommendation:**

 This is a well-conceived and rigorous protocol that addresses a very timely intersection of technology and behavioral health. The methodology is solid and aligns with current scoping review standards.

 The manuscript is scientifically sound as it stands, but the protocol would be significantly strengthened if the authors could:

 - Clarify the exclusion of qualitative research.

 - Acknowledge the inherent limitations of their grey literature search strategy.

 - Update the data charting form to explicitly capture ethical discussions.Addressing these points will provide a clearer roadmap before the review process officially begins.

Is the study design appropriate for the research question?

Yes

Is the rationale for, and objectives of, the study clearly described?

Yes

Are sufficient details of the methods provided to allow replication by others?

Yes

Are the datasets clearly presented in a useable and accessible format?

Not applicable

Reviewer Expertise:

I am a PhD candidate and research student specializing in Artificial Intelligence. My research focuses on using machine learning techniques to predict sporting events, manage betting risks, and optimize bettor portfolios.

I confirm that I have read this submission and believe that I have an appropriate level of expertise to confirm that it is of an acceptable scientific standard, however I have significant reservations, as outlined above.

# Response

**Published:** 2026-06-17

**Authors:** Ståle Pallesen

**Affiliations:** Dept of Psychosocial Science, University of Bergen, Bergen, Norway

This protocol sets the stage for a scoping review of AI applications within the gambling sector. As the authors point out, AI is already a staple of the digital gambling world—used for everything from harm detection to game design—yet we still lack a clear overview of its methodologies and ethical implications.

 The study is well-structured and follows established PRISMA-ScR and JBI frameworks. With a transparent search strategy and a pre-registered design, the methodology is both rigorous and reproducible. This work provides a much-needed foundation for anyone looking to understand the intersection of AI and gambling, from operators to policy makers.

**Critical Recommendations for Scientific Soundness**

 To further strengthen the protocol and the subsequent review, I suggest addressing the following points:

**1. Defining the scope of "AI Tools"** The inclusion criteria are quite thorough, but the mention of Large Language Models (LLMs) warrants more specific attention. Given the rapid pace of development in 2025, it would be helpful if the authors briefly discussed how they expect LLMs to appear in the gambling sector, perhaps in customer service bots or the analysis of player interactions. Specifically, how will these be categorized in the data charting form?

**RESPONSE:** Thank you for this comment. We have now added the following text to the introduction in order to more specifically address the potential role of LLM: « Furthermore, Large Language Models (LLM) have, for example, been utilized to respond to prompts regarding problem gambling. In this capacity, they can assist individuals in overcoming barriers (e.g., stigma) to seeking help, by offering an anonymous and judgment-free platform. ^40^ LLM also hold promise in detecting gambling issues within various online texts. ^41^«

**2. Enhancing the Data Extraction Plan (Table 2)**

**-Ethical Dimensions:** I recommend adding a dedicated field to Table 2 to capture whether the studies actually discuss ethics (e.g., privacy, algorithmic bias, or player exploitation). This is vital, especially since the introduction already highlights the ethical paradox of AI in this field.

**RESPONSE:** We have now added the following field: «-Ethics (e.g., privacy, algorithmic bias, or player exploitation)» to the AI dedicated fields in Table 2.

**-Performance Metrics:** To make the "key findings" more useful, the authors should ensure they consistently extract performance data like AUC, accuracy, or precision. This will make the final synthesis much more robust.

**RESPONSE: **We agree and have added the following in terms of key findings «(e.g., AUC, accuracy, precision)».

**-Refining Dissemination and Impact** While the dissemination plan is good, its impact could be improved by specifying how the findings will be tailored to different groups. For instance, clinicians might need a focus on risk-detection models, while regulators will be more interested in predictive behavioral modeling.

**RESPONSE:** Thank you for this suggestion. The sentences in questions now reads: «We will prepare a paper for submission to an open-access journal. The paper will present both risk-detection models, highly relevant for clinicians, and predictive behavioral modeling, which is of particular interest to regulators.”

**3. Recommendation:**

 This is a well-conceived and rigorous protocol that addresses a very timely intersection of technology and behavioral health. The methodology is solid and aligns with current scoping review standards.

 The manuscript is scientifically sound as it stands, but the protocol would be significantly strengthened if the authors could:

 - Clarify the exclusion of qualitative research.

**RESPONSE:** The following has now been added to the discussion in order to explain why qualitative studies will be excluded: «Qualitative studies will be excluded as the review aims to map quantifiable outcomes, statistical correlations, and numerical trends that fall outside the scope of qualitative methodologies.”

 - Acknowledge the inherent limitations of their grey literature search strategy.

**RESPONSE: **The following sentences have now been added to the discussion in order to acknowledge the inherent limitations of their grey literature search strategy: «To ensure the scientific rigor of the studies included in this scoping review, grey literature will be restricted to conference proceedings and dissertations. Still, this may leave out some relevant publications.”

 - Update the data charting form to explicitly capture ethical discussions. Addressing these points will provide a clearer roadmap before the review process officially begins.

**RESPONSE:** This has now been updated in the data charting form by adding the following: «-Ethics (e.g., privacy, algorithmic bias, or player exploitation)»
